# Exploring optimal group dynamics and their feasibility during a live-remote exercise program for cancer survivors: an international concept mapping study

**DOI:** 10.1007/s00520-026-11439-7

**Published:** 2026-11-07

**Authors:** Esther C. E. de Jongh, Anouk Hiensch, Laurien M. Buffart, David Binyam, Alina Kias, Karen Steindorf, Dorothea Clauss, Nadira Gunasekara, Yvonne Wengström, Poorna Anandavadivelan, Eva M. Zopf, Mark Trevaskis, Jon Belloso, Iñaki Ruiz-Pérez, Chloe Rezola-Pardo, Alberto Alves, Pedro Antunes, Catarina Garcia, Neil Aaronson, Anne M. May, Martijn M. Stuiver

**Affiliations:** 1 Department of Psychosocial Research and Epidemiology, Netherlands Cancer Institute, Amsterdam, The Netherlands; 2 Julius Center for Health Sciences and Primary Care, University Medical Center Utrecht, Utrecht University, Utrecht, The Netherlands; 3 Radboud University Medical Center, Nijmegen, The Netherlands; 4 Division of Physical Activity, Cancer Prevention and Survivorship, German Cancer Research Center (DKFZ) and, National Center for Tumor Diseases (NCT), Heidelberg, Germany; 5 Medical Faculty, University of Heidelberg, Heidelberg, Germany; 6 German Sport University Cologne, Cologne, Germany; 7 Division of Nursing, Department of Neurobiology, Care Sciences, and Society, Karolinska Comprehensive Cancer Center, Karolinska Institute, and, Karolinska University Hospital , Stockholm, Sweden; 8 Cabrini Cancer Institute, Cabrini Health, Melbourne, Victoria Australia; 9 Mary MacKillop Institute for Health Research, Australian Catholic University, Melbourne, Victoria Australia; 10 Biogipuzkoa Health Research Institute, San Sebastián, Spain; 11 GaituzSport Foundation, Bilbao, Spain; 12 Associação de Investigação de Cuidados de Suporte Em Oncologia, Vila Nova de Gaia, Portugal; 13 Research Center in Sport Sciences, Health and Human Development (CIDESD), University of Maia, Maia, Portugal; 14 National Program for the Promotion of Physical Activity, Directorate-General of Health, Lisboa, Portugal; 15 Department of Epidemiology and Data Science, AmsterdamUMC, University of Amsterdam, Amsterdam, The Netherlands

**Keywords:** Live-remote exercise, Cancer survivorship, Group dynamics, Group rehabilitation, Cancer rehabilitation, Symptom management

## Abstract

**Purpose:**

Live-remote supervised exercise helps cancer survivors reduce the negative side effects of their treatment, and group dynamics are an integral aspect of successful rehabilitation outcomes. In this international group concept mapping study, we investigated which aspects of peer interaction and trainer behaviour participants consider important and how feasible those preferences are according to exercise professionals.

**Methods:**

Participants who completed a 12-week live-remote exercise programme for cancer survivors were recruited in 5 European countries and Australia. Participants generated statements in response to prompts about patient interaction and trainer behaviour. The next group of participants sorted statements into thematically similar clusters and/or rated them by perceived importance. Exercise specialists rated the same statements according to feasibility. Data were analysed using multidimensional scaling, hierarchical cluster analysis, and qualitative interpretative analysis.

**Results:**

A total of 120 participants and 14 exercise specialists participated. The patient interaction prompt generated 45 statements organized into five clusters (mean importance rating 3.29; mean feasibility 3.61). The subjective experience of belonging to the group was the highest rated cluster for this prompt. The trainer behaviour prompt generated 56 unique statements organized into seven clusters (mean importance 3.82; mean feasibility 4.13). High-quality individual and group level instructions were rated as most important for this prompt.

**Conclusions:**

Participants and trainers agreed on the relative priorities, and all key aspects were rated as feasible, suggesting favourable conditions for implementation. Adequate instruction and supervision by trainers and a warm, welcoming atmosphere among participants were considered the most important aspects of live-remote training.

**Supplementary information:**

The online version contains supplementary material available at https://doi.org/10.1007/s00520-026-11439-7.

## Background

Exercise is increasingly recognized as an effective way to reduce symptoms and treatment-related side effects among cancer survivors, and initiatives to offer exercise through live-remote interventions are rapidly emerging [1–5]. Live-remote exercise interventions offer structured exercise to a target group, led by specialized trainers in real time through an online video conferencing platform at set times [3]. Although disparities seen in in-person supportive cancer care may still extend to online formats, such as in groups with a lower socioeconomic position, lower health or digital literacy, or language barriers—an online alternative can also reduce disparities by overcoming geographical barriers, concerns about infection risk, or discomfort due to symptoms [6–8]. Moreover, live-remote exercise programmes may be a more feasible first line of rehabilitation for individuals who are too deconditioned to travel to an in-person training session [2–8].

A growing body of evidence supports that live-remote exercise programs are effective, including in the cancer survivorship population [9, 10]. Yet, insights into participants’ preferences or expectations regarding social dynamics in this setting are much needed, as failure to meet such needs may limit intervention effectiveness and adherence [3, 11, 12].

In a rehabilitation context, the health care focus shifts from primary cancer treatment towards the patient’s physical and psychosocial functioning, as patients attempt a return to normal—a process closely related to an individual’s sense of identity and quality of life [13, 14]. Against this backdrop, an online exercise program constitutes a contextually rich environment in which patients interact with one another and the trainer through a digital setup. This includes unavoidably seeing others on screen and being seen through a webcam while training.

The group dynamic in live-remote exercise programs involves patient-to-patient interaction, enabling exchange with peers who have similar experiences while navigating cancer survivorship. However, patients may differ in their needs and preferences regarding contact with peers. The online context makes it difficult to withdraw from such interaction and simultaneously limits opportunities for informal supportive one-to-one side conversations among participants.

Patient–trainer interactions are shaped by expectations of tailored guidance, professionalism, and patients’ prior experiences with rehabilitation or exercise. Moreover, some patients may value recognition of the social implications of their treatment trajectory, including the loss of functioning, while others may, as cancer survivors, want to avoid being defined by their cancer diagnosis and treatment history [14]. To ensure these online formats are considered an appealing and accessible option when offered to a diverse survivorship population and to support adherence and maximize effectiveness, it is important to understand such preferences.

In this concept mapping study, we investigated how cancer survivors in five European countries and Australia prefer to interact with other participants, and what behaviours they prefer from the trainers in order to gain maximum benefit from live-remote training sessions.

Through end-user involvement, our aim was to (1) gain insight into the preferences of participants regarding patient-to-patient interaction and behaviour of the trainers; (2) measure how important they consider these collected preferences to be; and (3) assess how feasible these are to exercise specialists offering the program.

## Methods

This study was conducted parallel to the ongoing PREFERABLE-II LION RCT [15]. The LION-RCT aims to develop and evaluate the effects of a live-remote exercise programme for cancer survivors experiencing treatment-related side effects (i.e. emotional distress, fatigue, neuropathy, and/or reduced overall physical fitness). This exercise programme consists of three online training sessions per week over a 12-week period, with one training session targeting the most burdensome side effect [15].

### Participants

Participants of the PREFERABLE-II LION RCT at eight sites in six countries (Australia, the Netherlands, Germany, Sweden, Spain, Portugal) were invited to participate in the concept mapping study by local research staff immediately after completion of the 12-week live-remote exercise program without further in- or exclusion criteria [15]. Thus, participants in this study had completed primary cancer treatment including chemotherapy with curative intent between 3 and 15 months earlier and experienced at least one of the four target side effects before starting the live remote training: fatigue, neuropathy (chemotherapy-induced peripheral neuropathy), reduced physical functioning, or emotional distress. The exercise specialists who were invited to rate statements according to feasibility were eligible if they were part of the PREFERABLE-II Consortium and had hands-on experience with delivering exercise interventions to cancer survivors (either online or in-person).

We aimed to include as many participants per country as possible within the available time frame per study phase. Recruitment per site reflected the speed at which each country had recruited for the LION-RCT [15].

Although there are no formal sample size calculations for concept mapping studies, Rosas and Kane identified studies that included between 20 and 649 participants, with an average of 156 participants [16].

### Study design

Group concept mapping follows a structured, five-step procedure consisting of (1) statement generation in response to a focus prompt; (2) statement sorting according to likeness and rating according to perceived importance; (3) multidimensional scaling (MDS) of the sorted items to obtain a two-dimensional representation of the data—the point cluster map; (4) cluster analysis of this map; and (5) cluster interpretation and labelling [17–19]. The first two steps use an inductive, participant-driven approach, while steps 3–5 apply structured quantitative analysis and researcher interpretation.

Two focus prompts were developed during an in-person expert meeting, with all co-authors present, and special attention was given to ensuring the language was clear and easy to understand.

**A.** *What is important for you to benefit from interacting with other participants in your group?*

*To benefit from interacting with other participants, for me it is important that...*

**B.** *What behaviour of the trainer towards you do you find important during an online training programme?*

*During an online training programme, I find it important that the trainer…*

The final prompts were translated into the local language for all sites by consortium members.

### Data collection

We used the online concept mapping software GroupWisdom (Ithaca, NY) to enable data collection from multiple stakeholders independent of place and time [20]. Data collection began in October 2024 and ended in November 2025. The study fell under the ethical approval of the LION-RCT at all sites (trial no. NCT06270628; registered 2024-01−15).

#### Statement generation

Participants responded to both focus prompts sequentially, with support of a manual explaining the user interface and guiding them through the process. Some participants completed the brainstorm task at the respective institutes, and some completed the brainstorm task at home, yet they all used the online interface to do so. If participants completed the brainstorming phase on-site, researchers left the room while they responded to the prompts. For each prompt, participants could see the statements that had already been generated by others in their own language. Prior to beginning the task, they were encouraged to enter any statements they came up with, even if these were similar to what had already been recorded. Participants were told their trainers would not see the statements they generated.

#### Statement reduction

Statement reduction was performed using current guidelines and methods [21–23]. After the brainstorming phase closed (May 2025), all statements collected in non-English speaking countries were translated into English. Two native-speaking researchers at each site translated the statements in parallel and compared results to reach consensus. In an idea synthesis/statement reduction meeting (May 2025), four researchers (EJ, AH, LB, and MS) reviewed all statements per theme and removed duplicates. In case of duplicates, the most representative statement for one idea was chosen and reworded for clarity, if needed. The statements containing multiple ideas were separated to ensure each statement contained a single idea. The separated statements were coded so that they could be retraced to their original iteration.

This process resulted in 45 statements for patient interaction (A) and 56 statements for trainer behaviour (B). The final set of statements was translated back into each site language by the native-speaking researchers and uploaded to the GroupWisdom interface.

#### Sorting and importance ratings

The next group of participants were asked to sort the previously generated statements into clusters of related ideas and/or to rate statements according to importance. Identical to the brainstorming phase, participants who completed the 12-week training program were invited on a rolling basis. To reduce participant burden, participants generally either sorted and rated the statements for one of the prompts (A or B) or rated the statements for both prompts (A and B).

For the sorting task, participants were asked to sort statements that they perceived to be related thematically into piles, and name the piles accordingly, using the graphical user interface of GroupWisdom. They were instructed not to sort piles into statements that were ‘important to them’ or ‘unimportant to them’, but they could organize statements in any other way that made most sense to them. For the rating task, participants were asked to rate the perceived importance of each statement on a 5-point Likert scale (5 = most important).

#### Feasibility ratings

Participating exercise specialists were instructed to rate the same statements according to feasibility. As statements were formulated in the first person to be most accessible for patients, we instructed the exercise specialists to be mindful of this and to rate statements from an implementation and research perspective. The 5-point rating scales ranged from *not at all feasible* (1) to *entirely feasible* (5). When rating statements for feasibility, exercise specialists were unaware of the corresponding participants’ importance ratings per statement, and vice versa.

## Data analysis

We considered sorting data entries with more than 25% of statements left unsorted to be incomplete due to premature termination of the task, consistent with the cutoff programmed into GroupWisdom, and therefore did not extract these data entries manually. All sorting and rating data were assessed for quality. Data of participants who sorted statements according to a value rating (e.g. importance) were considered unusable (*n* = 3), and obviously uniform ratings, indicating careless or insufficient effort responding, were removed from rating data (*n* = 1) [24, 25]. Cluster titles as assigned by the participants were translated into English at all non-English sites by researchers from the respective sites. Finally, the sorting and rating data from six countries (including translated labels) were combined into a single file.

Scaling and cluster analysis were done in R (version 4.5.3; package: R-CMap) [26, 27]. First, multidimensional (ordinal) scaling was applied to the similarity matrix. Goodness of fit for the resulting point-cluster map is expressed as a stress value between 0 and 1, with values below 0.37 considered an acceptable fit [16, 17]. Next, using hierarchical cluster analysis, the statements on the point map were grouped into clusters based on their Euclidean distance. We decided on the appropriate number of clusters through visual interpretation of the dendrogram and consideration of what made conceptual sense, striving for the simplest solution without combining two distinct themes in a single cluster (MS, EJ). The descriptive label for each cluster was developed (EJ, MS) based on the statements in each cluster and the cluster names given by participants during the sorting exercise.

## Results

In total, 120 participants from The Netherlands (40%), Portugal (13%), Spain (9%), Germany (9%), Australia (8%), and Sweden (4%) were included across all phases.

Fourteen exercise specialists from all six countries contributed to the feasibility ratings. Participant descriptives are reported in Table 1.

**Table 1:** Participant information per task and topic

**Task**		**Patient interaction**	***N***		**Trainer behaviour**	***N***
**Brainstorm**	**Total *N* = 38**			**Total *N* = 45**		
	**Sex**	*Female*	33	**Sex**	*Female*	36
		*Male*	5		*Male*	9
	**Age category**	*Under 50 or 50*	10	**Age category**	*Under 50 or 50*	12
		*51–60*	14		*51–60*	18
		*61–70*	9		*61–70*	10
		*71–80*	5		*71–80*	5
	**Country**	*Portugal*	10	**Country**	*Portugal*	10
		*Spain*	8		*Spain*	5
		*The Netherlands*	8		*The Netherlands*	19
		*Germany*	6		*Germany*	5
		*Sweden*	2		*Sweden*	2
		*Australia*	4		*Australia*	4
**Sorting**	**Total *N* = 20**			**Total *N* = 26**		
	**Sex**	*Female*	17	**Sex**	*Female*	20
		*Male*	3		*Male*	6
	**Age category**	*Under 50 or 50*	7	**Age category**	*Under 50 or 50*	6
		*51–60*	8		*51–60*	9
		*61–70*	4		*61–70*	6
		*71–80*	1		*71–80*	5
	**Country**	*Portugal*	6	**Country**	*Portugal*	2
		*Spain*	2		*Spain*	2
		*The Netherlands*	7		*The Netherlands*	16
		*Germany*	0		*Germany*	2
		*Sweden*	2		*Sweden*	0
		*Australia*	3		*Australia*	4
**Rating**	**Total *N* = 38**			**Total *N* = 32**		
	**Sex**	*Female*	31	**Sex**	*Female*	23
		*Male*	7		*Male*	9
	**Age category**	*Under 50 or 50*	14	**Age category**	*Under 50 or 50*	8
		*51–60*	13		*51–60*	11
		*61–70*	8		*61–70*	7
		*71–80*	3		*71–80*	6
	**Country**	*Portugal*	7	**Country**	*Portugal*	3
		*Spain*	5		*Spain*	6
		*The Netherlands*	11		*The Netherlands*	17
		*Germany*	6		*Germany*	3
		*Sweden*	5		*Sweden*	0
		*Australia*	4		*Australia*	3

**Participants between categories are not mutually exclusive. Total number of unique participants = 120**

The feasibility ratings for both topics were completed by a heterogeneous group of exercise specialists, including physiotherapists (general, sports, oncology, and lymphedema specialists), exercise physiologists (clinical and oncology-focused), sports therapists, personal trainers, yoga instructors, nurses with fitness training, and researchers in exercise oncology.

The exercise specialists had a broad range of clinical experience, with the largest groups having 3–6 years (*n* = 4) and 13–18 years of experience (*n* = 4). The majority did have prior experience with online supervised interventions before being involved in the LION-RCT (*n* = 9) [15].

### Clusters: patient interaction

**Fig. 1:**
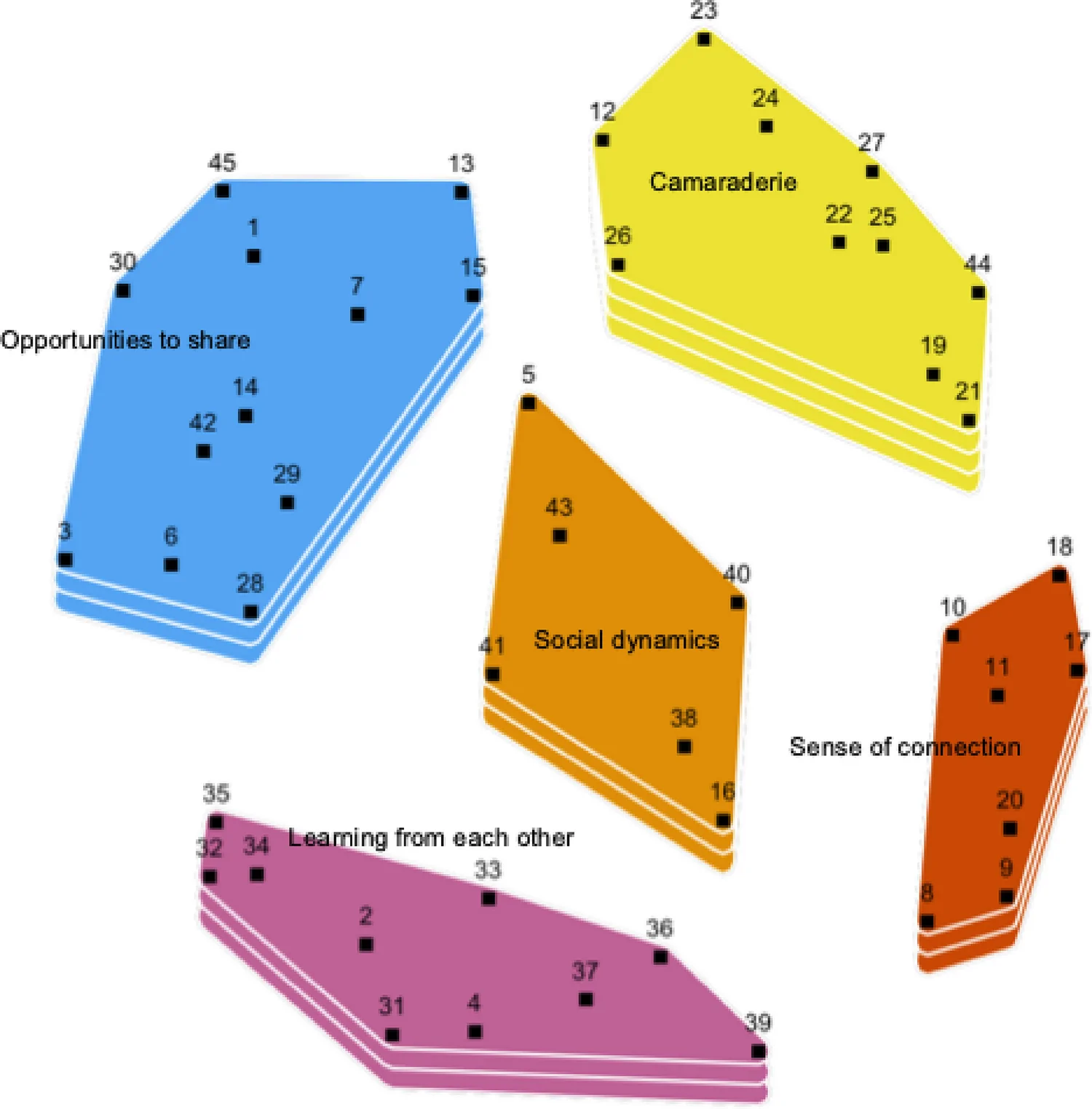
Cluster rating map for statements about interaction with other patients. Patient interaction: clusters with smaller dimensions on the spatial map indicate greater agreement between participants’ sorting. The number of layers per cluster indicate the absolute mean importance rating of the statements in that cluster

The point map for **patient interaction** had a stress value of 0.218. We considered five clusters to be the optimal solution to organize the statements about interaction with other patients (Fig. 1). Online resource 1 contains statements generated in response to the patient interaction prompt, organized by clusters.

### Statements: patient interaction

**Fig. 2:**
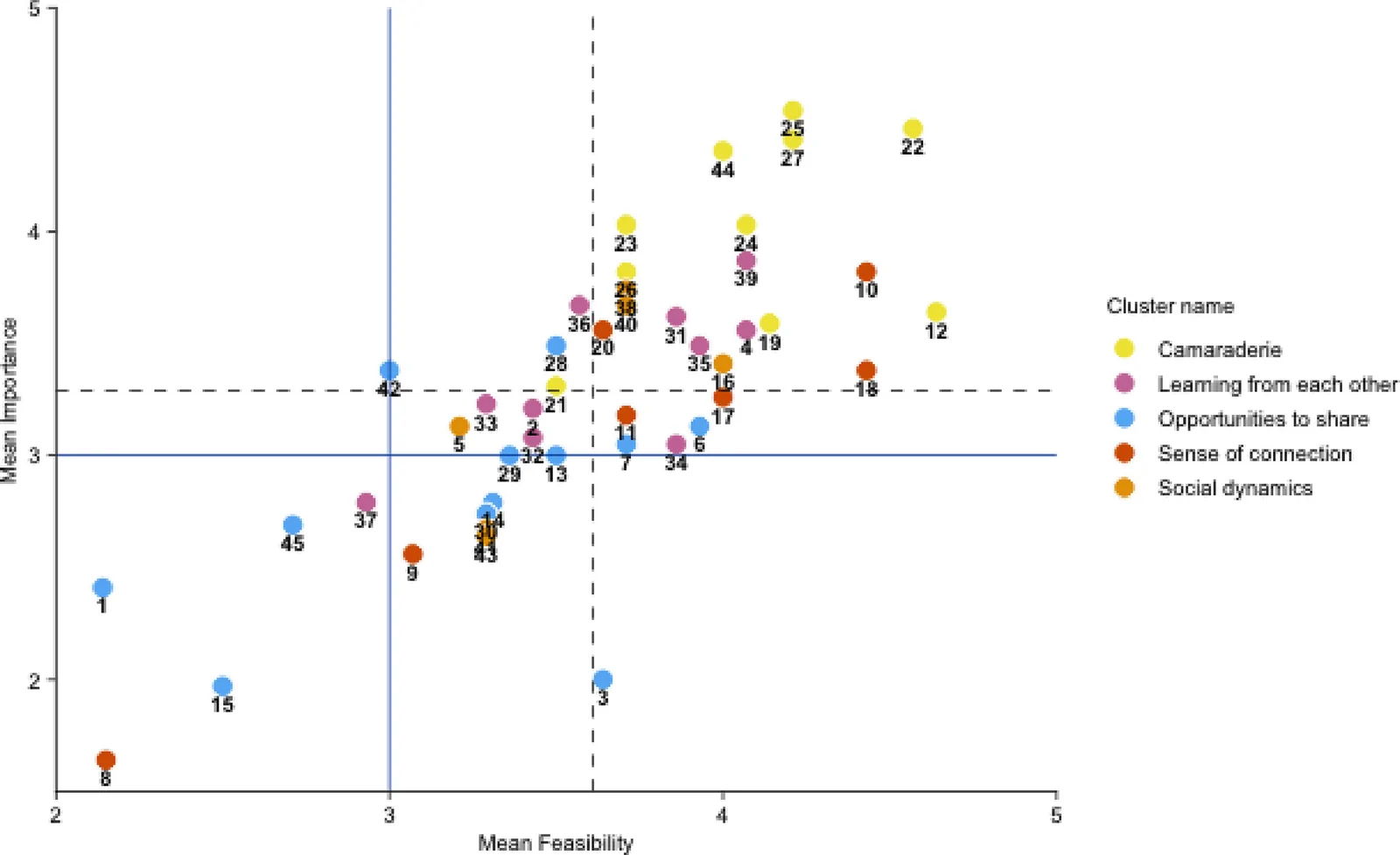
Go-zone map including statements about patient interaction. This bivariate graph shows the mean ratings for each statement’s importance as perceived by participants (*y*-axis) and feasibility as perceived by exercise specialists (*x*-axis). The solid blue line reflects the neutral ratings (3 on a scale from 1 to 5). The black dotted axis lines dividing the quadrants reflect the mean importance and feasibility ratings of all statements. Accordingly, statements in the right upper quadrant are considered above average for importance and feasibility. Numbers indicate statements.

The mean importance ratings of all statements for patient interaction ranged from 1.64 to 4.54, and their feasibility ratings ranged from 2.15 to 4.21. The statements in the cluster *Camaraderie* were almost exclusively rated as above average for importance and feasibility, as opposed to the statements in the cluster *Opportunities to share*, of which all statements were rated as below average for importance and feasibility or even below neutral (Fig. 2).

### Clusters: trainer behaviour

**Fig. 3:**
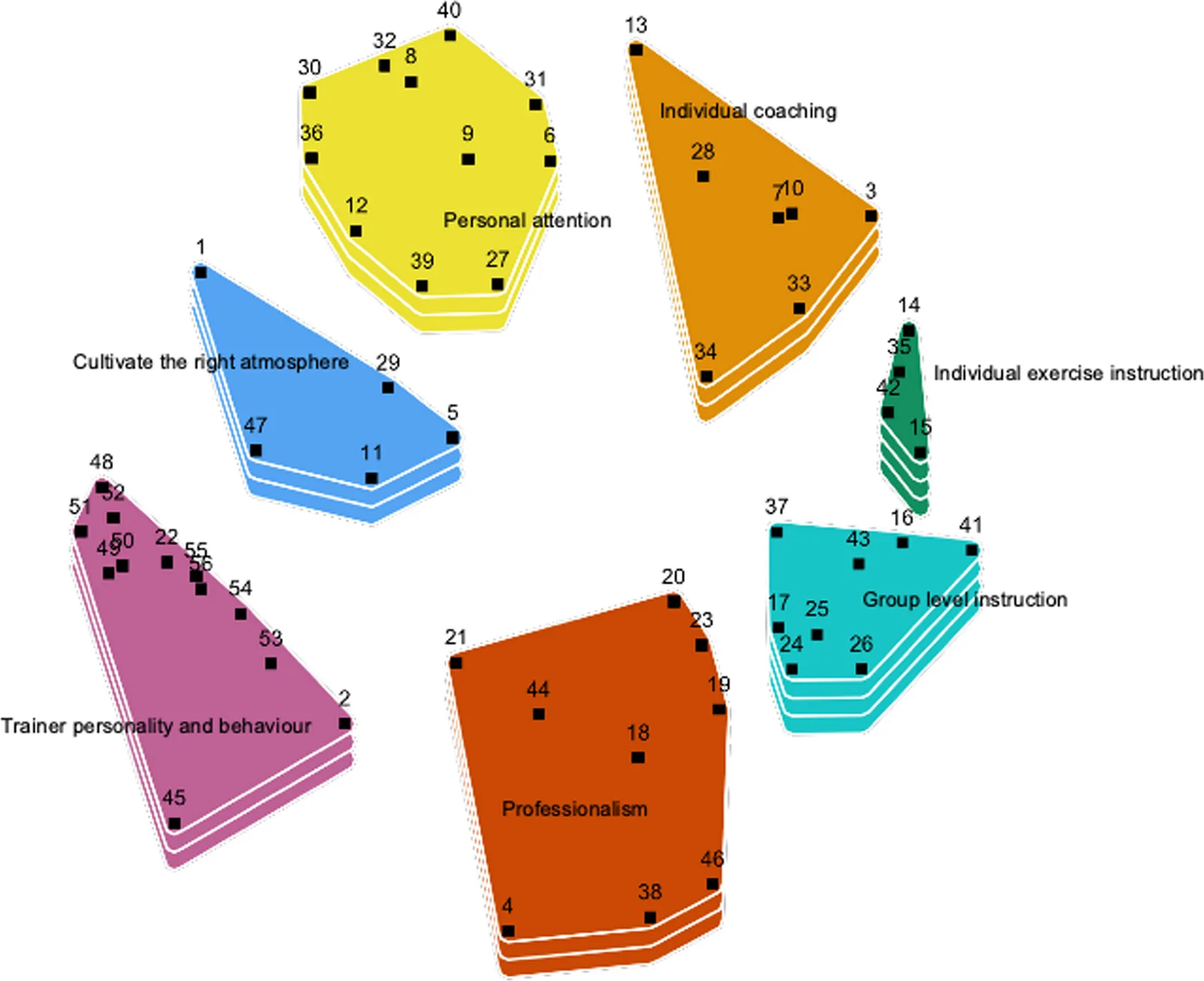
Cluster rating map for statements about preferred behaviour of trainers. Trainer behaviour: clusters with smaller dimensions on the spatial map indicate greater agreement between participants’ sorting. The layers per cluster indicate the absolute mean importance rating of the statements in that cluster

The point map including statements about **trainer behaviour** had a stress value of 0.197. Seven clusters were considered optimal to organize the statements within this topic (Fig. 3). Online resource 1 contains statements generated in response to the trainer behaviour prompt, organized by clusters.

### Statements: trainer behaviour

**Fig. 4:**
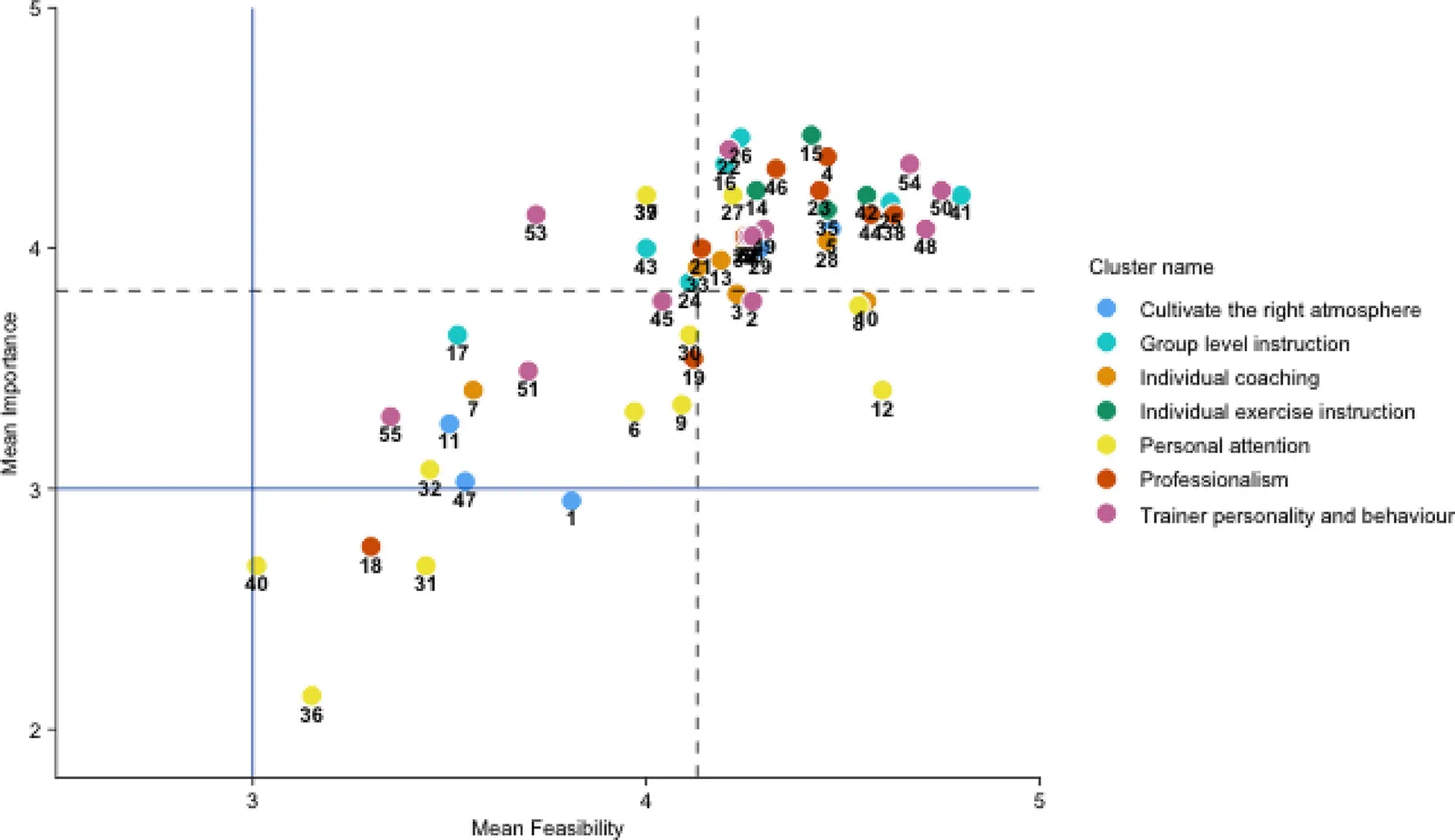
Go-zone map including statements about trainer behaviour. This bivariate graph shows the mean ratings for each statement’s importance as perceived by participants (*y*-axis) and feasibility as perceived by exercise specialists (*x*-axis). The solid blue line reflects the neutral ratings (3 on a scale from 1 to 5). The black dotted axis lines dividing the quadrants reflect the mean importance and feasibility ratings of all statements. Accordingly, statements in the right upper quadrant are considered above average for importance and feasibility

The mean importance ratings of all statements for trainer behaviour ranged from 2.14 to 4.47, and their feasibility ratings ranged from 3.15 to 4.42. The statements in the cluster *Individual exercise instruction* and *Group level instruction* were predominantly rated as above average for importance and feasibility. The statements in the cluster *Personal attention* and *Cultivate the right atmosphere* were, on average, rated as less important by participants (Fig. 4). Even within the ‘below average feasibility’ side of the go-zone map, statements were still rated above or close to the neutral line (blue), indicating that all statements regarding trainer behaviour were considered feasible to implement by exercise specialists.

**Fig. 5:**
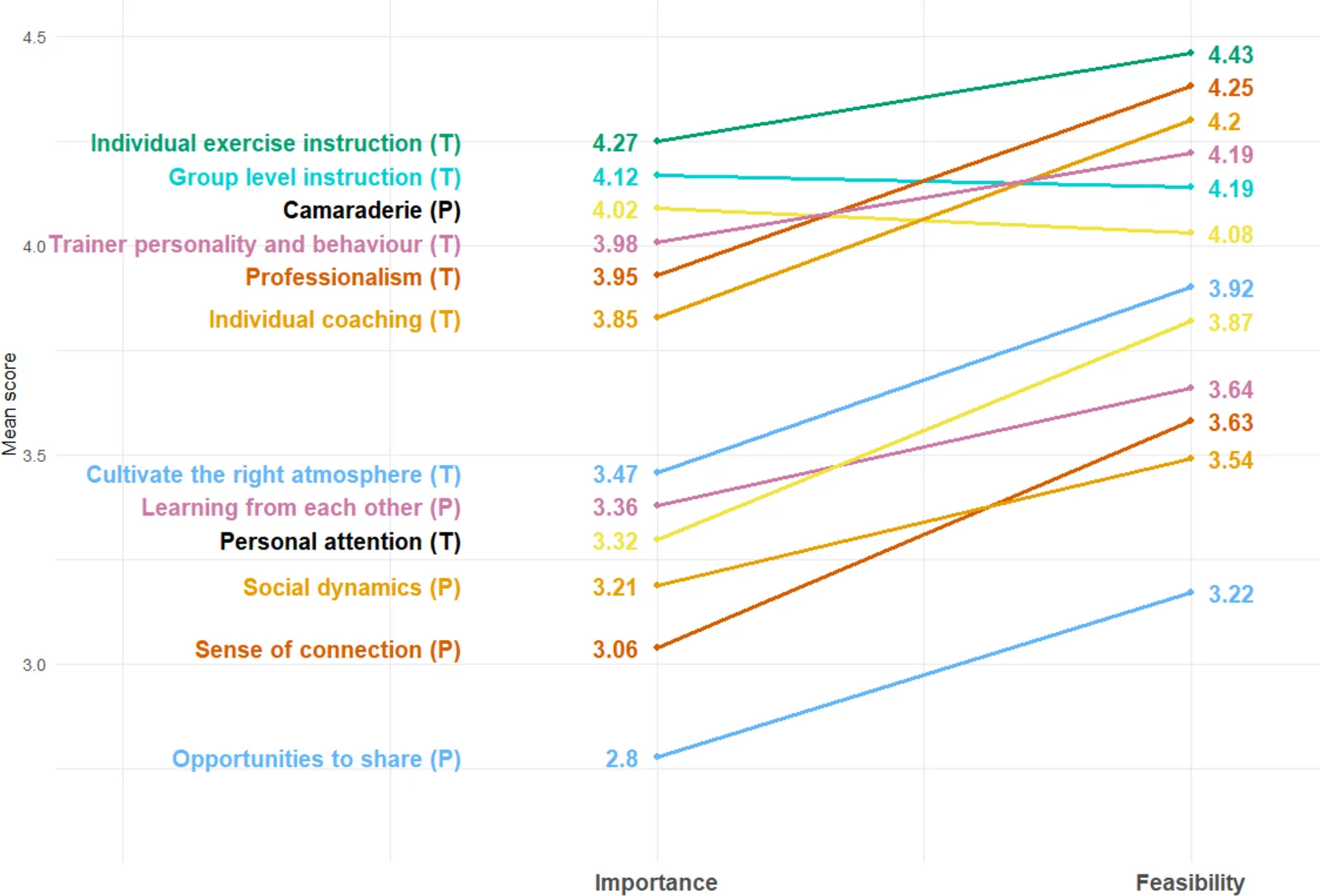
Pattern match: mean importance vs. mean feasibility. Pattern match for all clusters, comparing the mean importance (left) versus how achievable they are (mean feasibility; right)

Among all clusters combined, the clusters rated as most important belong to the trainer behaviour prompt (e.g. individual exercise- and group-level instruction)—except for Camaraderie, which was rated among the top clusters as well (Fig. 5).

## Discussion

To the best of our knowledge, this is the first international concept mapping study investigating what cancer survivors who completed a 12-week live-remote training program prefer from their interaction with peers and the behaviour they expect from trainers in order to benefit most from the program. In addition, this study is distinct in being the first to quantify which aspects of group dynamics in live-remote training for cancer survivors are considered both important by participants and feasible by exercise specialists. Previous studies have examined acceptability of or general experiences with an online exercise intervention [28, 29], and studies on related topics collected data through interviews [29–31] or included participants with no disclosed prior experience with online exercise interventions [30, 32, 33]. Almost all clusters rated as most important fell under the trainer behaviour prompt, except for *Camaraderie*, which includes statements generated in response to the patient interaction prompt, and was rated as the third most important cluster by participants overall (Fig. 5). This indicates that, besides the ability for trainers to deliver personalized and adequate instruction and guidance to the individual and the group, participants consider it a priority that they experience a sense of belonging and a positive and cheerful atmosphere with their peers. All issues considered important to patients were also considered feasible by exercise professionals. This finding is reassuring for implementation, as it indicates that the quality of group dynamics does not have to suffer in an online environment.

Pattern matching of feasibility and importance showed that, generally, statements related to trainer behaviour were rated as both more important and feasible than the statements within the patient interaction topic. Regarding feasibility ratings, this is likely because, as specialists and developers of the program, we have much greater control over how trainers act than over how participants interact with one another. The higher importance ratings suggest that patients expect more from their trainers than from their peers in an online program.

In response to the patient interaction prompt, *Opportunities to share*, which includes statements about opportunities and reasons to share information or experiences and reflects preferences for anonymity or awareness of others in the group, was rated as the least important and least feasible concept. However, the statements in this cluster are still relatively important and should be facilitated whenever possible. *Camaraderie* was the highest rated patient interaction cluster, and among the highest rated clusters overall. *Camaraderie* includes statements about feeling a sense of belonging and experiencing a warm and welcoming group atmosphere. The cluster about a related topic within the trainer interaction prompt, *Cultivate the right atmosphere*, was not rated as highly, suggesting that participants rely on their peers more than the trainers for a positive atmosphere and group feeling. Two other clusters within the patient interaction prompt also related to the concept of *Camaraderie*, are *Social dynamics* and *Sense of connection*, but are distinct in the sense that they are more about objective characteristics and practical conditions of the group (e.g. size, demographics, which behaviour occurs) rather than what it ‘feels’ like to be in the group, which is more difficult to plan for. However, the high importance ratings of *Camaraderie* indicate that it is not sufficient to pay attention to group composition only, but that trainers supervising online programmes should learn how to actively cultivate group dynamics and create an atmosphere in which there is a positive and welcoming dynamic among peers. Creating a warm and socially cohesive atmosphere is important for more reasons than achieving participant satisfaction; social support and identification with the group are the most important predictors of successful changes in health-related behaviour in groups [34–36]. As such, trainers are recommended to foster group cohesion by focusing on group dynamics strategies such as shared leadership, team-building exercises, and collective goal setting during the group trainings [37–39].

Among the clusters about trainer behaviour, *Personal attention* and *Cultivate the right atmosphere* were rated as least important and least feasible. The cluster *Individual exercise instruction*, a cluster with four statements relating to instruction and guidance adjusted to the individual, was rated highest on both scales, indicating that patients value provision of adequate individual- and group-level instruction over everything else, and that this is a priority that is easily achieved.

Previous qualitative studies exploring general experiences and preferences during online rehabilitation initiatives, similarly demonstrate that patients consider a welcoming, supporting, and warm environment to be important [29, 40]. Consistent with findings by Dennet et al. participants in our study also prioritized a trainer’s ability to adjust exercises to their current ability (cluster: *Individual exercise instruction*) and a trainer’s ability to provide variation and alternative exercises for different levels (cluster: *Group level exercise instruction*) [40]. Studies using a concept mapping design remain scarce, and have so far only been conducted in the context of in-person exercise and its effect on quality of life in patients with cancer [5], or the implementation of exercise in routine cancer care [41].

Although pertaining to in-person settings, findings from a pilot randomized trial suggest that group-dynamics strategies may support physical activity maintenance among cancer survivors [37]. A related systematic review found that most group-based exercise interventions did not describe or test group-dynamics strategies, concluding that it remains unknown how to foster and optimize group dynamics [42]. This concept mapping study addresses this gap in the context of live-remote exercise, offering a structured account of which elements of group dynamics participants consider most important (and trainers consider feasible) to benefit from these programs.

### Study limitations and strengths

This study included participants of the LION-RCT exercise program, which means their responses to the respective phases of this study were derived from their experience with this program [15]. Participants might have responded to the focus prompts either based on elements they experienced as positive during the online trainings (i.e. aspects that were present and memorable), or on elements they perceived as missing. Although we did not systematically record whether responses were grounded in experienced versus absent components of the training, it is likely that both are reflected in the data. Furthermore, the voluntary nature of participation in this study likely further limited representativeness of the sample, i.e. by including those with strong opinions on this topic. On the other hand, people who feel less strongly about the topic are also less likely to be affected by group dynamics that do not fully suit their preferences. The study included fewer Swedish participants compared to the number of participants from the other countries, as recruitment for the LION-RCT began later in Sweden and inclusion for this study was directly dependent on the speed at which patients were being included in the LION-RCT. Although the sample size did not allow for comparisons between countries, which was beyond the scope of the study, we have no reason to believe this relative underrepresentation impacted the results. Our sample was also limited by an underrepresentation of men and older individuals (71+), which did not allow for meaningful subgroup analyses by sex or age. Future research with a larger and more balanced sample would be valuable to explore whether these factors influence the relative importance of clusters.

A strength of the study was the asynchronous inclusion of multiple stakeholders based in six countries, resulting in rich and multicultural data. Also, including feasibility ratings by expert specialists enabled the formulation of workable recommendations for future initiatives in this field. The concept mapping design does not facilitate an in-depth exploration of the individually brainstormed statements. However, the combination of our chosen platform and the comprehensive mixed methods of analysis resulted in insights that pertain to the needs and expectations of individuals during live-remote training after cancer treatment specifically.

## Conclusion

The results of this international group concept mapping study indicate which aspects of group dynamics should be prioritized in live-remote exercise interventions for cancer survivors: providing supervision by trainers with strong competencies in providing high quality exercise instructions in the online setting; and subjective feelings of belonging to the group and creating a welcoming atmosphere. Although cancer survivors indicate that they rely more on their peers than on the trainer to bring about the latter, it is important to note that exercise specialists also considered fostering this aspect of group dynamics as highly feasible. Participants and trainers agreed on the relative priorities of all clusters, and all clusters were rated as feasible, pointing to favourable conditions for implementation. The findings of this study can be used to optimize interaction and social dynamics of live, remotely supervised exercise programs.

## Supplementary information

Below is the link to the electronic supplementary material.

ESM 1 (PDF 125 KB)

## Data Availability

Data is available upon reasonable request.
