# Supplementary material for: Exploring optimal group dynamics and their feasibility during a live-remote exercise program for cancer survivors: an international concept mapping study
Source: Support Care Cancer. 2026 Nov 7;34(12):1208. doi: 10.1007/s00520-026-11439-7 (PMC13744539; doi:10.1007/s00520-026-11439-7)
Supplement: Supplementary file 1 [file 520_2026_11439_MOESM1_ESM.pdf]

Online Resource 1

Statements generated in response to the **patient interaction** prompt, organized by clusters.

Cluster name	Importance	Feasibility	Number of statements in cluster
Camaraderie	Mean 4.02; SD = 0.42	Mean 4.08; SD = 0.37	10
	12. We know each other's names<br>19. I feel like I am part of the group<br>21. I feel a sense of belonging with like-minded people<br>22. We are friendly and kind to each other<br>23. We can laugh with each other<br>24. We have fun together<br>25. The atmosphere is positive<br>26. The contact is and remains casual<br>27. I feel at ease<br>44. We feel comfortable indicating when we reach our limits		
Learning from each other	Mean 3.36; SD = 0.34	Mean 3.64; SD = 0.38	10
	2. The exercise program includes an in-person training session<br>4. We don't engage in small talk for too long, but focus on exercising together<br>31. We can share ideas on how to continue exercising after the program has ended<br>32. We can share tips about exercises or physical activities outside of the classes<br>33. We can share tips about the exercises performed during the training session<br>34. I can receive tips from people who have more experience with the exercise program because they started earlier<br>35. We can share tips about improving physical fitness and wellbeing<br>36. We motivate and encourage each other while training<br>37. We challenge each other while training<br>39. We improve our physical fitness together		
Social dynamics	Mean 3.21; SD = 0.48	Mean 3.54; SD = 0.32	6
	5. We can talk during the training session<br>16. Everyone attends as much as possible<br>38. We motivate each other without putting pressure on each other<br>40. Everyone is actively participating, even if it not everything goes smoothly<br>41. We don't talk too much about the illness<br>43. We see each other's strengths and weaknesses		
Sense of connection	Mean 3.06; SD = 0.74	Mean 3.63; SD = 0.81	7
	8. I train in a group with people of the same gender<br>9. I train in a group with people who are similar to me with respect to, for example, age and ability<br>10. The groups are small<br>11. I mostly train with the same group of people<br>17. I am exercising with people who are going through the same illness<br>18. I am exercising with people who are also rehabilitating<br>20. The participants have a shared sense of purpose		
Opportunities to share	Mean 2.8; SD = 0.48	Mean 3.22; SD = 0.53	12
	1. We meet in-person at least once outside of the online training sessions<br>3. I don't know which kind of cancer someone has<br>6. There is some time before or after the sessions to share experiences<br>7. We have some time to talk<br>13. We get to know each other<br>14. I know if others are enjoying it as much as I am<br>15. I know why someone is not attending		

	28. We can share experiences on coping with physical and mental problems<br>29. We have time before or after the classes to share experiences and help each other to better cope with the illness<br>30. We can share our feelings and personal lives with each other<br>42. We are open with each other about the complaints we have<br>45. We have an opportunity to socialize outside of the classes

Statements generated in response to the **trainer behaviour** prompt, organized by clusters.

**Cluster name**	**Importance**	**Feasibility**	**Number of statements in cluster**
Individual exercise instruction	Mean 4.27; SD = 0.14	Mean 4.43; SD = 0.12	4
	14. Tells me whether I am doing the exercises correctly<br>15. Corrects my posture or movement<br>35. Adjusts exercises to my current ability<br>42. Respects it when I can't do an exercise and looks for an alternative way of performing it		
Group level instruction	Mean 4.12; SD = 0.27	Mean 4.19; SD = 0.39	8
	16. Pays close attention to whether the exercises are performed correctly<br>17. Offers variation in the exercises<br>24. Explains the purpose of the exercises<br>25. Demonstrates the exercises<br>26. Gives a clear and precise instruction of the exercise<br>37. Delivers the training in a way that accommodates everyone<br>41. Can provide alternative exercises if someone is impaired<br>43. Adapts the pace to ensure everyone can complete the exercise		
Trainer personality and behaviour	Mean 3.98; SD = 0.33	Mean 4.19; SD = 0.43	12
	2. Starts on time<br>22. Communicates well<br>45. Is patient when technical difficulties occur (e.g., poor internet connection)<br>48. Is empathetic<br>49. Is friendly<br>50. Is respectful<br>51. Has a sense of humor<br>52. Is enthusiastic<br>53. Makes the class fun and enjoyable<br>54. Has a positive attitude<br>55. Is spontaneous<br>56. Is patient		
Professionalism	Mean 3.95; SD = 0.51	Mean 4.25; SD = 0.4	9
	4. Has good technical equipment and connection<br>18. Provides variation in the background music<br>19. Takes part in the exercises with us<br>20. Keeps the other trainers informed about my progress, if there are multiple trainers<br>21. Works well in a team with other trainers<br>23. Explains the training sessions well<br>38. Is knowledgeable about the symptoms and side effects of cancer treatment<br>44. Is patient when demonstrating or explaining the exercise<br>46. Has expertise in training people diagnosed with cancer		
Individual coaching	Mean 3.85; SD = 0.22	Mean 4.2; SD = 0.32	7

	3. Tells me when I am not clearly visible and provides recommendations on how to adjust my camera<br>7. Notices when I'm struggling with the exercises and calls me later to provide advice<br>10. Assesses before each training whether it is safe for me to join<br>13. Reassures me when things aren't going as well<br>28. Challenges me to improve<br>33. Knows which equipment I use<br>34. Is responsive to personal needs and health problems		
Cultivate the right atmosphere	Mean 3.47; SD = 0.54	Mean 3.92; SD = 0.44	5
	1. Doesn't engage in small talk for too long before starting the training session<br>5. Answers any questions that participants may have<br>11. Promotes group interaction<br>29. Provides encouragement<br>47. Is open about his/herself, which encourages others to open up as well		
Personal attention	Mean 3.32; SD = 0.65	Mean 3.87; SD = 0.53	11
	6. Provides an opportunity for a private discussion<br>8. Checks how everyone is doing at the beginning and end of every training<br>9. Asks how the previous training session went<br>12. Praises me when I do well<br>27. Motivates me to keep going<br>30. Asks me about my wellbeing<br>31. Pays attention to what is going on in the participants' home/training environments (in the background)<br>32. Knows a little about my difficulties even if they have nothing to do with physical health<br>36. Pays attention to personal events like birthdays<br>39. Is able to attend to each individual in a group setting<br>40. Knows about my social background		
